# A systematic review on the physical, mental, and occupational effects of exercise on pregnant women

**DOI:** 10.1016/j.dialog.2024.100181

**Published:** 2024-05-12

**Authors:** Nathalia Rodrigues-Denize, By Tara Rava Zolnikov, Frances Furio

**Affiliations:** a National University, Department of Community Health, San Diego, CA, USA; b California Southern University, School of Behavioral Sciences, Costa Mesa, CA, USA; c California State University, Sacramento, CA, USA

**Keywords:** Pregnancy, Yoga, Resistance training, Aerobic training, Aquatic activities, Physical activity, Dance, Running, Walking, Cycling

## Abstract

Complications in pregnancy have been proven to be less frequent with exercise. The American College of Obstetrics and Gynecology suggests pregnant women should exercise an average of 20 to 30 min a day; however, only 13% of pregnant women exercise throughout their pregnancy. This amount could be improved if women are aware that exercise: 1. Can help avoid pregnancy complications or death and 2. Comes in various forms with different health outcomes associated with it. For this reason, this systematic literature review was conducted to review different types of exercise for pregnant women. Peer-reviewed articles were selected to discuss the benefits of the most researched exercises for pregnant women, which included yoga, resistance training, aquatic exercises, dance, and aerobic exercise such as running, walking, and cycling. Data from the review revealed that different types of exercises led to various benefits at different stages of pregnancy. Aquatic activities and yoga helped pregnant women toward the end of their pregnancies, whereas resistance training, dance, and aerobic exercises provided the most benefits during the first trimester. Other studies found that any form of exercise for 30 min a day or every other day for 60 min provided extensive beneficial results. Ultimately, this information could be used to create individualized exercise plans for pregnant women to adhere to throughout their pregnancy.

## 1 Introduction

There are approximately 211 million pregnancies that happen per year with 123 resulting in successful birth [1]; these rates are not likely to decrease, with over 50% of women expecting to have a child in the future [2]. That said, 1.2 million babies die from stillbirth and 2.9 die in the first month because of premature birth or complications during birth [1]. The leading causes of these deaths largely occur from hemorrhaging, cardiovascular, and coronary conditions [3].

Physical changes occur, which can contribute to complications in pregnancy or childbirth. Pregnancy modifies all systems in the body to ensure adequate fetal growth and development, such as excessive weight gain, moving bone structures, and hormonal changes [4]. Another modification is a woman's changing adaption of a metabolic state, which increases insulin resistance [4]. If not monitored and treated, physical and mental outcomes can have negative effects. For example, obesity can lead to gestational diabetes, hypertension, and depression, which can result in pregnancy complications [5,6]. In the United States, 12 to 25% of pregnant women reported being overweight and approximately 25% reported being obese [5]. Depressive symptoms rise up to 25% more during pregnancy and a decrease in gestational weight gain has been shown to also alleviate these symptoms [6].

Fortunately, most symptoms during pregnancy can be regulated, prevented, and significantly decreased with physical activity [7]. For example, exercise has been found to lower the risk of all-cause mortality by 31% [8], reduce gestational diabetes mellites by 30% [9] and decrease cardiovascular complications by 30% [10]. However, only 14% of pregnant women in the United States achieved moderate activity and only 23% had some type of vigorous activity [11]. Heskett and Everson (2016) also found that only 12 to 45% of women exercise at all, by standards developed by the American College of Obstetrics and Gynecology [12].

Improving exercise in women needs to be more clearly defined. For example, if a woman thinks of “exercise” as running 5 miles, she may not want to do it. If “exercise” is presented as an online, easily accessible, and free yoga class, then women may be more apt to attend, take part in it, and integrate it into their lives. More specifically, if a pregnant woman is diagnosed with gestational diabetes, then a more individualized exercise regime (e.g. more like a needs-based than an enjoyment-based approach) could be created and established for her to improve health outcomes. This information suggests a need to determine best practices for pregnant women to encourage exercising, while also understanding which form of exercise can be used to reduce pregnancy symptoms. Research has been conducted on women and each type of exercise; however, comparing each type of research to create a workout guide that best benefits pregnant women has not been conducted.

### 1.1 Purpose of the Study

Research indicates women who exercise can reduce the side effects of pregnancy, resulting in a healthier delivery, and a healthier baby weight [13]. A significant amount of research has been conducted on exercise and pregnant women; however, nothing is currently available that compares the benefits of different forms of exercise in pregnant women. The purpose of this systematic review is to provide information on what type of exercise as well as guidelines that are best for pregnant women. The expectation of this research is to home in on themes and patterns that can help establish best practice guidelines for women to follow during the different stages of their pregnancy.

## 2 Methods

This systematic review was conducted in July of 2020 and February 2024 on published articles discussing pregnant women, different forms of exercise, and their effects. A systematic review must be methodological, explicit, comprehensive, and reproducible [14]; the review must explain procedures that were conducted as well as include all relevant material concerning the topic discussed [14]. The review must meticulously scan all articles published on the topics discussed to find answers to the research questions [15], which in this case, were the following: Which exercise leads to the best results during a pregnancy? Is one type of exercise better during a specific time in pregnancy?

The search engines used to access scholarly articles include Google Scholar, ProQuest, EBSCOHost, PubMed, ScienceDirect, Elsevier, and the National Center for Biotechnology Information (NCBI). The Center for Disease Control and Prevention (CDC) was also utilized to uncover current statistics on pregnancy and exercise. These organizations were also used to discover prevalent pregnancy complication issues. The key terms used to search the databases for suitable articles include “pregnant women and exercise or physical activity and benefits”, “pregnant women and resistance training”, “pregnant women and aquatic exercise”, “pregnant women and running”, “pregnant women and walking”, “pregnant women and yoga”, pregnant women and complications”, “pregnant women and aerobic exercise”, “exercise”, “pregnant women and physical complications”, “pregnant women and dance”, “pregnant women and cycling”, “pregnant women and dance therapy”, “pregnant women and bicycling”, and “pregnant women and mental health issues”.

**Fig. 1:**
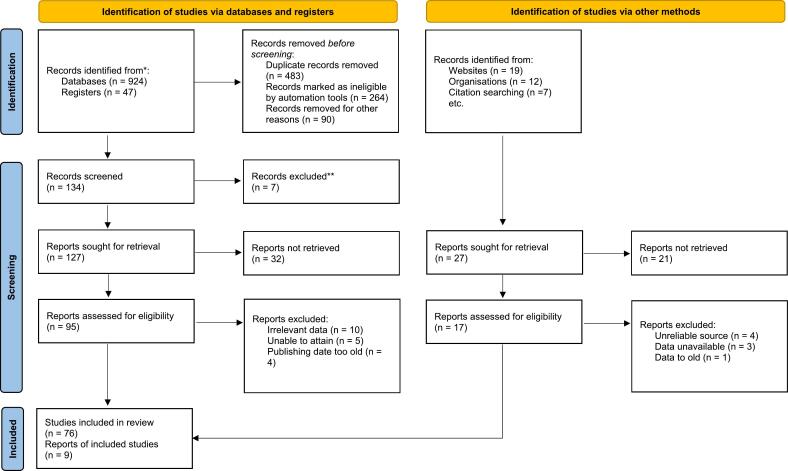

The goal of an analysis systematic review is to diminish bias by identifying, synthesizing, and appraising all pertinent studies for the specific topic, pregnant women and exercise [16]. There are eight steps to creating a systematic review and these are: formulate the review questions, define inclusion and exclusion criteria, develop a search strategy and find studies, select studies, extract data, asses study questions, analyze and interpret data, and disseminate findings [16]. Data analysis in a systematic review began by devising a question to review, defining questions, and creating a hyposithesis [16]. The next step was to determine the type of study design, which in this case was a systematic review [16]. The third and fourth step included running search engines to discover articles related to the topic studied and select relevant studies to review extensively [16]. In this case, a Preferred Reporting Items for Systematic Reviews and Meta-Analyses (PRISMA) diagram was used to identify inclusion and exclusion criteria for data Fig. 1. The PRISMA diagram aims to help researchers and authors improve the collection and reporting of a systematic review [17]. The articles selected were based on pertinent content regarding the effects of exercise on pregnant women. The fifth step was to extract the significant data from each article discovered to establish connections and reliability [16]; this was accomplished by using tables to organize information, such as Table 1, which helped data management. The next step was to determine if the data discovered was quality data to be analyzed and interpreted to determine the relevance of the article in relation to the topic studied [16]. This was achieved through peer review, wherein each researcher reviewed the articles and agreed upon inclusion. The eighth and final step is to publish a condensed version of the article at hand [16]. These processes were followed by the researchers to construct a comprehensive article with significant information that can assist with future research.

**Table 1:** Some articles indicating increase and decrease in physical outcomes during pregnancy

Physical Activity and Outcomes: ↓ = Significant Decrease ↔ = Insignificant Difference ↑ = Significant Increase															
Sources	Pregnancy Symptoms											Design	Method		
	Anxiety/Depression	Hypertension	GDM	Preeclampsia	Preterm Birth	Labor Duration	Maintain weight	Active Labor Pain	Body Pain	Cesarian Delivery	Other	Type of Exercise	Study	Sample Size	Gestation Age (weeks)
Campbell & Nolan, 2018	↓											Yoga	Qualitative	22	15–34
[18]								**↔**	↓	**↔**		Yoga	Mixed or Other	X	X
[19]						↓		↓	↓	↓		Yoga	Quantitative	350	14–40
[20]		↓		↓								Yoga	Mixed or Other	X	X
Rakhshani, 2012				↓								Yoga	Quantitative	68	12–28
[21]						↓						Yoga	Qualitative	52	14–40
[22]									↓			Aquatic Activity	Qualitative	11	20–32
Bachi et al., 2018					↓							Aquatic Activity	Quantitative	140	8–39
[23]					↓				↓			Aquatic Activity	Mixed or Other	X	X
[24]									↓			Aquatic Activity	Quantitative	11	30–34
[25]		↓							↓			Aquatic Activity	Quantitative	773	9–40
[26]		**↔**	**↔**	**↔**	↓							Aquatic Activity	Quantitative	32	1–40
[27]					↓							Aquatic Activity	Quantitative	66	20–40
Prewitt-White et al., 2019	↓											Resistance Training	Qualitative	22	1–40
[28]									↓			Resistance Training	Qualitative	17	15–40
[29]		**↔**							**↔**	↓		Resistance Training	Quantitative	92	14–25
[30]							↓					Resistance Training	Quantitative	X	X
[31]										↓		Resistance Training	Quantitative	X	X
[32]			↓	↓								Resistance Training	Mixed or Other	X	X
[33]										↓		Resistance Training	Qualitative	284	1–40
Gonenc & Dikmen, 2020	↓											Dance	Quantitative	93	38–42
[34]	↓											Dance	Mixed or Other	X	X
Verdin et al., 2020	↓								↓	↓		Dance	Quantitative	5	13–40
Daniel et al., 2015		↓	↓									Dance	Quantitative	34	X
Haakstad & Bo, 2011							**↔**					Dance	Quantitative	105	X
[35]	↓											Walking	Qualitative	200	36–40
[36]			↓	↓	**↔**		↓					Walking	Mixed or Other	X	X
[6]					**↔**					↓		Walking	Quantitative	1293	1–40
[37]			**↔**									Walking	Quantitative	24	20–40
[38]	↓					↓		↓				Running	Mixed or Other	X	X
[39]	↓											Running	Quantitative	110	1–40
[6]					↓		↓	↓		↓		Running	Quantitative	1293	1–40
[40]							↓					Running	Mixed or Other	1177	1–40
[36]								↓		↓		Cycling	Mixed or Other	X	X
[41]			**↔**									Cycling	Quantitative	12	30–40
O'Neil, 1996					↓							Cycling	Quantitative	11	34–37
[42]			↓				↓					Cycling	Mixed or Other	X	X
[43]											↑	Multiple Exercise	Mixed or Other	X	X
[44]											↑	Tennis	Mixed or Other	X	X
[45]											↑	Tennis	Quantitative	18	12–42
[46]											↓	Gymnastic	Mixed or Other	X	X
[47]	↓											Gymnastic	Quantitative	132	18–22
Granath et al., 2016									↓			Gymnastic	Quantitative	390	11–40
[48]		↓										Gymnastics	Quantitative	6	18–40

## 3 Results

Exercise and pregnancy were overall positively connected and contributed to improved health benefits for pregnant women; the top ten types of exercise for pregnant women were discussed. Overall, walking or running was found to have the most significant decrease in pregnancy symptoms that arose specifically because of exercise. That said, different exercises such as yoga, aquatic exercises, and dancing were found to help pregnant women with mental health, while aerobic exercises and resistance training helped women more so with physical health. An interesting finding was that exercise appeared to improve outcomes for pregnant women in an occupational or professional context. The results from each article was further expanded to discuss significant decreases or insignificant differences based on the type of exercise and health outcome [Table 1].

### 3.1 Yoga

Yoga is the practice of stretching and strength-building without the use of tools or machines. Yoga is typically practiced for an hour a day for about three times a week. Each session is held one to three times a week for 50 to 60 min [18]. The most benefits occurred when practiced for 10 to 12 consecutive weeks or more [18]. For pregnant women, guidelines advised against any yoga movement that caused tightening around the abdomen [49]. Additionally, pregnant women should avoid any positions that require lying on their back or any back bending positions [49], inversions (e.g. handstands), and advanced positions that can harm the body and fetus [49]. Yoga positions, such as triangle pose, bound angle pose, cat pose, warrior pose, forward fold, squatting, and retaliation pose, should be modified throughout a pregnancy [50]. For example, the final resting pose should be performed on a surface where one's head and back can be elevated to reduce potential pressure on major veins [50]. Yoga can be a safe way to exercise with the use of modification to help ease pregnancy complications.

Approximately 7% of women practice yoga throughout their pregnancy^21,^ though yoga has been shown to positively influence mental aspects such as anxiety, depression, anger, and stress [51]. Yoga decreases depressive and anxiety symptoms in pregnant women who practice it [18,52] as well as mediate some physical and physiological complications that pregnant women may experience, such as lower back pain, leg pain, and pelvic pain [18,53]. Akarsu et al. [54] noted how yoga practices can be utilized toward the management of pregnancy symptoms [54]. For example, the use of stretches and relaxation caused a significant decrease in lower body pain [18]. Additionally, regular pelvic muscle exercise through postural yoga can increase the tone of pelvic muscles helping decrease active labor and maternal pain [19]. Furthermore, yoga increased parasympathetic activity and reduced sympathetic activity in the third trimester, which sequentially decreased hypertension and preeclampsia [20]. Moreover, another study further confirmed significantly less incidents of preeclampsia for women who practice yoga during pregnancy [55].

However, there are inconclusive results regarding yoga practice and duration of active labor. In one study, pregnant women were found to have a shorter duration of labor; however, the difference was statistically insignificant [18]. Yet, a decrease of two hours in labor was also discovered in a different study [21]. Tejwani et al. [19] found a 7.16% increase in prolonged labor for women as well as 26.19% higher rate of emergency cesarean section in women who did not practice yoga or discontinued yoga throughout their pregnancy [18,19]. A study by Schmidt et al. [56] also demonstrated reduced cesarean delivery in related to physical activity [56].

### 3.2 Aquatic Activities

Aquatic exercises involve any type of movement performed in the water [57]. Research has proven aquatic exercises can lead to a decrease in joint overload through the help of buoyancy in water activities [22]. The average apparent weight of a pregnant woman in water is reduced by nearly 83% when immersed in water up to the xiphoid process [24]. Moreover, because pregnant women are more susceptible to injury due to imbalance, water activities encourage physical movement while avoiding harsh falls [24,23].

Aquatic activities and water emersion were also found to decrease lower back, edema, and pelvic pain [25,26]. A statistically significant difference was not found regarding blood pressure rates [26], but another study did discover a decrease in hypertension incidence amongst pregnant women who exercised using aquatic activities [26]. Pregnant women were found to have fewer false contractions and a longer gestation period when exercising in the water [3,[22], [23], [24],^26^,^27^].

Aquatic guidelines seemed to follow suit with most other types of exercises. Weights in water could be used to create drag or resistance to help build muscles [23]. Aquatic yoga, noodles, and a kickboard could be used in the water to improve stability and flexibility [23]. Most studies also kept an average pool temperature of 83 °F to 86 °F to avoid extreme cold or warm temperatures to prevent overheating or hypothermia [4,57,22,23]. Moreover, the best results were found in studies where women exercised 45 to 60 min long, three times a week, for 12 to 28 weeks [57,22,23]. Most sessions included an eight to ten minutes warm-up, a 30-min freestyle, and a 10 to 12-min cooldown. Another guideline is to utilize exercises such as frontal kicks, butt kicks, and stationary running [24]. These exercises were conducted at 15 repetitions with a five-minute rest in between each of the two sets [24]. Additionally, establishing a session that would include four 100-m laps as a warmup with six Aqua Mama exercises also provided beneficial results [22]. Finally, it should be noted that there was no significant difference found between water activities compared to land activities [3].

### 3.3 Resistance Training

Resistance Training is the third most common type of exercise that women participate in while pregnant [58] and is as any form of training that increases muscle strength using weights or resistance bands. Only 10% of pregnant women have reported engaging in this type of exercise due to its extreme nature [58]. With this exercise, women reported an increase in motivation, improved mood swings, and reduced stress throughout pregnancy [58,29]. More research focuses on the physical benefits of the exercise. Benefits included a decrease in nausea, fatigue, and headaches [28], and possible decreased blood pressure [29]. Improved outcomes included decreased preeclampsia rates by 24% and decreased gestational diabetes by 59% [32]. One study suggested that more women gave birth naturally without the need for medication [31], while two other studies did not notice a significant difference in modes of delivery [29,59]. A different study also confirmed no significant differences between complications during birth while using resistant training [32]. Although most studies did not find a significant difference between variables, all studies found this type of exercise did not lead to any adverse effects throughout the pregnancy on the women or child. Interestingly, a case study of a pregnant women who trained for 18 total weeks found increased lean muscle mass with higher repetitions and lower weights were utilized [30]. This breakthrough led to the assumption that RT can help maintain maternal bodyweight throughout pregnancy. In addition to weight maintenance during pregnancy, women who practiced resistant training returned to their pre-pregnant weight within two weeks to three months after birth [28].

Resistance training guidelines should be followed strictly as this type of exercise can cause injury. One of the main guidelines is to train one's core muscles to stretch instead of contract, similarly to yoga [32]. Most exercise sessions were held for 60 min three times a week. Training every non-consecutive day helped increase neuromuscular recuperation [32]. Moreover, as a pregnancy progresses, modifications to resistant training exercises should also occur. During the first trimester, no modifications need to occur, except using proper form and avoiding ballistic movements [32]. In the second and third trimester, pillows and towel use can assist with certain movements [32]. The supine position should be avoided, which can obstruct the venous return from the uterus resulting in a lack of oxygen [31]. Additionally, two to three sets of 12 to 15 repetitions per set of each exercise should be done, with rest for two to four minutes between each set [31]. The idea is to perform a high amount of repetition with a low weight amounts [32]. Pregnant women can use free weights that are preferably no more than five pounds [32]. Moreover, lunges, still leg deadlifts, jumping and squats should be avoided [31,60]. Additionally, it is best to exercise after meals to avoid hypoglycemia [32].

### 3.4 Dance

Dance is the expression of oneself through body movement [61]. Dance has been increasingly recognized in healthcare as a form of physical activity due to its multiple benefits [61]. Dancing has been shown to hep pregnant women create positive feelings and emotions regarding their pregnancy [62]. Additionally, like yoga, dance or dance therapy has been found to decrease depression and increase energy levels for pregnant women [34]; these effects can also decrease anxiety during pregnancy [63]. Dance has also been found to help maintain blood pressure by decreasing systolic and diastolic (down 11%) blood pressure [64]. Another study by Santos and colleagues (2005) observed an 18% increase in cardiorespiratory fitness in 12 weeks of exercise three times a week [65]. The same program was replicated by Halvorsen and colleagues in 2013, who reviewed oxygen uptake between pregnant women who danced versus those who did not but found no statistically significant differences between the groups [65]. There was no statistically significant association regarding maternal weight gain [65]. One emergency cesarean and no miscarriages were discovered during a study regarding dancing through labor [63]. Additionally, there was a significant decrease in pain compared to women who did not dance while in labor due to a decrease in pelvic floor activation which caused a distraction from pain associated with labor [63]. Some movements that can help with labor pain include circular dance movements of the pelvis and waist, pelvic tilts, and dancing to the left and right [65]. Most pregnant women indicated dancing helped them feel more control and less fearful over their labor experience [63].

Dancing guidelines can help create a safer environment for pregnant women to exercise. Jumping movements should be avoided [63,65]. Additionally, having chairs around to help once maintain their balance and avoid the risk of falling is another guideline that can encourage safe dancing [63].

### 3.5 Aerobic Exercise

A variety of studies demonstrate the benefits of exercise on maternal health [81]. The most common type of exercise during pregnancy was aerobic exercises [59]. Aerobic exercises are known to improve and maintain physical fitness amongst pregnant women [42]. The recommendations per exercise by the American College of Obstetrics and Gynecology usually focuses on aerobic exercise benefits for pregnant women [38]. Some aerobic exercises include running, walking, or cycling.

### 3.6 Walking

Walking is a rhythmic and dynamic process that uses large muscle groups from the legs, limb-girdle, and trunk [66]. About 85% of pregnant women who exercise report walking as their choice of exercise [33]. Moreover, walking is also perceived to be the safest and easiest physical activity for a pregnant woman [67]. There is a statistically negative correlation between depression and daily walking, and as daily walking increased, depression and anxiety levels decreased [35]. This is significant because a decrease in depressive symptoms has been found to reduce anxiety symptoms [35]. Other health benefits include a 4–21% decrease in glucose levels after walking, thus leading to a 20% decreased risk of gestational diabetes and a 33% decrease risk of preeclampsia [36]. However, no correlations were found between the HbA1C levels and walking [68]. Other research found a 29 to 44% decrease in risk for weight gain outside normal limits in pregnant women who walked [36]. Moreover, walking has been shown to ease some pain associated with active labor. There was also a decrease for gestational age at delivery [32]; but it is still unclear on the relationship between walking and preterm birth, which means walking cannot be attributed to delivery dates [36]. Assisted vaginal delivery rates increased [32], but an association between walking and cesarean delivery risk was not found [36]. Moreover, walking half an hour a day lead to a decrease of 0.551 pounds in gestational weight [36]. Although a significant relationship could not be determined in gestation age, walking can also help decrease postpartum weight retention [36].

Walking guidelines are one of the easiest to abide by because walking does not require any form of equipment. A total of 10,000 steps is required to enhance benefits [36]. A steps study was further created by to determine a positive outcome of approximately 11,060 steps daily [68]. Guidelines were also given to exercise on a treadmill to avoid inconsistent terrain as well to create a safer environment [67]; except that hills on the treadmill should be avoided with warming up at a lower mile per hour advised [67]. Another suggestion was to focus on the quality of one's exercise instead of the distance of one's walk, which can contribute to an accomplished positive feeling [37]. Focusing less on steps and more on time, another study determined that an average of 25 to 40 min a day can help decrease blood sugar levels [36]. Despite the step-count, time, or pace, all types of walking were found to be beneficial for pregnant women.

### 3.7 Running

Running can be an intense, weight-bearing activity; therefore, it is usually considered secondary to walking as a physical exercise for pregnant women [32]. An increase in mental health, as well as emotional support, is linked to an increase in running amongst pregnant women [59]. Pregnant women who run tend to have a heightened awareness of the changes occurring in their bodies during their pregnancy, which can help them connect with their pregnancy [38,40]. Additionally, women who ran during their pregnancy also tended to be less likely to report postpartum depression [39]. Running caused an increased oxygen uptake [38], which may lead to an increase in circulation and blood flow to the fetus and the body [38]. Nevertheless, a decrease in oxygen consumption (VO_2_ max) was found in women who ran at 32 weeks than at 20 weeks [67]. Additionally, another study concluded that women who never ran throughout their pregnancy gained an average of 5 pounds compared to women who did run [32,40]. Moreover, a 29–44% decrease risk for weight gain outside the recommended amount for pregnant women who ran was also determined [36]. Further research suggested that benefits of running while pregnant, such as a decrease in triglyceride levels and a resting heart rate, were apparent [38]. Running also creates a stronger pelvic floor muscle, thereby possibly contributing to an improved labor process [38]. There was no significant difference in gestational age at delivery or birthweight percentile [32]. Therefore, trends toward an earlier delivery or a lower birth weight have been debunked [32]. However, the vaginal delivery rate increased significantly by 25% in women who ran [32]. Some women even stated they felt running made the labor and delivery go faster [38].

Running guidelines should be created to help decrease the overexertion of a fetal heart rate. Therefore, maintaining distance regularity during one's pregnancy, instead of running for distance, can benefit the fetus and woman [38]. Additionally, pregnant women should focus on the quality of the run and their associated feelings [38]. The talk test can determine if a pregnant woman is overexerting oneself during exercise [42]; if one can carry a conversation, then the pregnant woman is performing an adequate amount of exertion upon the body [42]. Moreover, running for 30 min to 60 min for two to seven days a week found the most benefits during pregnancy [42]. Guidelines that can help women decrease the risk of falling by running on a treadmill and wearing proper or correct running shoes should be encouraged [38]. A pregnant woman's feet tend to change in the arches as well as flatten or widen during pregnancy [38], so proper shoe wear can help mitigate avoidable injuries [38]. Lastly, pregnant women should stop running around 27 to 28 weeks [69]. These weeks tend to be the time as to when most pregnant women feel pelvic pressure from running [69]. Adhering to guidelines can help pregnant women enjoy running while also avoiding risks to the fetus and the body.

### 3.8 Cycling

Cycling has not been as predominately advised as walking or running however, recent research has started to support cycling as a type of physical activity for pregnant women [70]. Cycling is commonly known as a non-weight bearing exercise characterized by the engagement of the cardiovascular system as well as skeletal muscles [71]. Cycling can also serve as a rehabilitation program that can be ideal for pregnant women [71]. Little to no articles were discovered regarding mental health benefits while cycling. One article noted interval cycling while pregnant led to more pregnant enjoying exercise than continuous cycling [41]. During a study in 1996, a significant difference in maternal heart rate was not discovered [72]; this may lead one to debunk the concept that cycling may increase a pregnant woman's internal temperature thus overheating one's body. Another study regarding the difference between cycling in a supine position versus an upright position found an increase in blood and pulse pressure while cycling in a semi-supine position [70]. An increase in heart rate and oxygen consumption in conditional cycling and a 28% increase in energy expenditure in interval cycling was also discovered [41]. However, a difference in blood glucose was not found. Finally, cycling can reduce weight gain as well as the risk of gestational diabetes [42]. Additionally, a gestation age of 264 days and 288 days was also notated [72], which means that cycling does not increase early delivery. Furthermore, babies were both in the 10th and 90th percentile for weight, debunking the notion that cycling may lead to a small gestation weight [72].

More research studies should be performed to increase proper guidelines for cycling while pregnant. Most guidance suggested an average of 30 min about three times a week from week 13 to week 37 weeks, which would lead to potential benefits for cycling [42]. Pregnant women should avoid repetitive sit and stand motions throughout a cycling class, and instead, simply sit while cycling. Additionally, one should attempt to cycle in an upright position to avoid an increase in pulse pressure [73]. Upright cycling positions were also found to lead to safer cycling experiences [74]. Bennett [74] also stated that women should use wide seats to better increase comfortability [74], while another study recommended that pregnant women should cycle on a stationary bicycle to avoid the risk of falls [41]. Guidelines for cycling—especially heated cycling classes—throughout pregnancy should be further researched as cycling can be dangerous.

## 4 Discussion

This systematic review sought to answer how different types of exercise benefit pregnant women. This question was answered by targeting articles chosen to enhance the relevance of this topic. Perhaps the most reasons to highlight this research is that only about 14 to 20% of women exercise while pregnant and most drastically reduce their exercise once pregnancy begins or shortly thereafter [75]. Despite the positive outcomes associated with pregnancy, it is still not actively being considered and practiced during pregnancy. The goal of this review was to discuss different types of exercise and establish ideas for workout guidelines for pregnant women to follow. This information can help prevent health complications throughout one's pregnancy and create a sense of motivation and perseverance [58].

One key finding was that each type of exercise provided unique results. While exercise all together provided positive effects, yoga provided predominantly mental health benefits, including stress and depression reduction [51]. Yoga also largely helped with relaxation and breathing practices for an easier and more natural labor process [21]. This type of exercise also helped create appropriate posture, balance, stability, and spinal alignment results in less hip and pelvic pain [49]. That said, these physical benefits were also matched with dance and dance therapy. Dance therapy helped with an easier labor process as well as a decrease in labor pain [63]. Similarly, aquatic exercises also provided unique benefits to a pregnant woman, as this type of exercise helped improve outcomes from women who were more likely to suffer complications. This is because aquatic exercises helped decrease edema in a pregnant woman and thermoregulate one's body [57]. Aquatic activities were also the best form of exercise for women who were near the end of their pregnancy; the buoyancy provided by the water helped decrease the workload on a pregnant woman's joints [24]. Resistance training provided different benefits to a pregnant woman as well; ultimately, resistance training helped increase maternal weight management and have leaner masses as well as stable body fat [32,30]. Aerobic exercise, such as walking, running, or biking, contributed to decreased blood pressure, gestational diabetes, preeclampsia, and glucose levels as well [42,76]. Walking, an easily accessible form of exercise that does not require additional equipment, could be used during each trimester of pregnancy [38]. All this information highlights the benefits of various types of exercises during pregnancy.

That said, there are some basic guidelines that need to be followed while exercising when pregnant. For example, woman should not lie on their back after 20 weeks nor should they perform any inverted exercises such as handstands [31]. This guideline was specifically advised in articles discussing resistance training and pregnant women as well as yoga and pregnant women. Most articles also discuss overexerting oneself while performing high-risk exercises, such as boxing [77]. Moreover, uneven terrains should be avoided during aerobic exercises, such as walking, running, or biking to prevent falls [67]. Some articles provided specific exercises for pregnant women such as butt kick for five sets of 15 repetitions. A higher repetition was accompanied by a lower amount of weight regarding resistance training and aquatic activities [32]. A recommended time limit for exercise was 30 min each day or 45 to 60 min every alternate day, with the second option being preferred to allow muscle recuperation [42]. The saying, “a little goes a long way” can be used in this context as well, suggesting that a small amount of exercise a day should be performed with a goal of reaching 150 min a week [42].

Combination exercise was also reviewed. Many articles found mixing two or more forms of exercise created more favorable outcomes [6,78,25,79]. For example, land and aquatic exercises was shown to work together in preventing gestational diabetes [25]. Moreover, combining resistance training and aerobic exercise provided advantageous outcomes as well [33]. In general, most studies confirmed the benefit for warmup and cooldown routine; for example, an eight to ten-minute warmup and an eight to ten-minute cool down or stretching could improve health outcomes [60,67]. To replace this, a short yoga class could be practiced, which also includes stretching, relaxation, and learn breathing techniques. One program created a 20-min aerobic exercise, 20 to 25-min resistance straining, and a 10-min stretching workout plan [76]. This may add to an exercise workout plan or routine to create more favorable outcomes during pregnancy because of the diversity of exercise and use of different muscle groups. The combination of dance and walking tended to help decrease casual glucose levels [64], which may be more common in the second and third trimester. Exercise and effects can occur during the length of the pregnancy or at specific times. Aquatic spinning or cycling, aquatic yoga, and equating resistance training can all be part of a pregnant woman exercise routine during the third trimester, so can be held consistent across the pregnancy [23]. This type of information can be used to layer solutions for women who face various health effects during pregnancy. For example, first trimester could be running and yoga to support strength, weight management, and stretching, but as the pregnancy progresses, aquatic exercise and walking could be used instead. This information offers a flexible guide to help encourage exercise in pregnant women, to achieve maximum health outcomes for both them and their child.

Most research articles in this systematic review debunked the previous notion of reducing prolonged levels of physical exertion during pregnancy [75]. In fact, repetition of information in all selected articles increased the ability to delve into a conclusion regarding exercise during pregnancy. The literature review also highlighted other factors, such as creating a combination of exercise for pregnant women to participate in at different stages of their pregnancy or who suffer from specific ailments (e.g. blood pressure).

This review did have some limitations. There were limitations to keyword searches and search engines used to find articles for pregnant women and different forms of exercise. A few full-text articles requested were not processed; therefore, hindering some possible connections throughout the literature review. This may have led to some inconsistent results. Some other limitations within the studies included small sample sizes and lack of pregnant women populations (e.g. those who need childcare or transportation, etc.). Moreover, most articles did not analyze or track the dietary habits of individuals, which could also contribute to adverse or improved symptoms experienced by pregnant women. Additionally, most studies involved voluntary participants, thereby creating a selection bias with certain populations. Lastly, the dropout rate due to incomplete surveys, incomplete interventions, discomfort, or miscarriages was not taken into consideration in most studies. Finally, some articles also did not consider the level of fitness a woman had before pregnancy.

Some other exercises during pregnancy were reviewed; however little to no articles were discovered. Articles states to avoid activities that increase the risk of loss of balance and fetal trauma such as horseback riding, water and snow skiing, collision sports such as basketball, tennis, or soccer, gymnastics, and hanggliding [43,44]. Tennis has also not recommended for pregnancy due to its increase risk in falls, tennis has also been found to increase bone mineral loss in pregnant women [45]. Another type of exercise to avoid while pregnant includes scuba diving. Scuba diving has been shown to increase the risk of decompression sickness and the potential for a miscarriage [43]. Additionally, gymnastics has been shown to enhance the overall tone of the body and the mobility of the hip joints [46] as well as a decrease in stress and anxiety [47]. Additionally, during normal pregnancy, practicing gymnastics should not be more than 40 to 45 min at least 2 to 3 times a week [46]. While gymnastics is recommended, avoiding twist and jerks is a must [46]. Additionally, in the untrained athlete, intrauterine fetal development did slow and should be avoided [46]. While precautions should be taken during land gymnastics, aquatic gymnastics has been shown to decrease low back pain or pelvic girdle pain [80]. In fact, a decrease in resting heart rate and BP were both discovered in pregnant women who practiced water gymnastics versus those who exercised on land [48].

## 5 Conclusion

The effects of pregnancy can be difficult for women but having the proper tools to avoid complications (e.g. gestational diabetes or nausea) can create a more positive situation. Exercise can help decrease the Walking appears to have the most benefits throughout the entire pregnancy because of its low-cost maintenance; walking also does not involve a lot of jumping and jerking movements and, therefore, can be performed throughout the entire pregnancy. Running, walking, cycling, and resistance training are more beneficial at the beginning of pregnancy, whereas aquatic activities, dance, and yoga offer more health outcomes toward the end of pregnancy. Combining different forms of exercise, such as yoga, resistance training, aerobic exercise, and aquatic activities, are ideal and can target physical ailments of pregnant women. That said, many forms of exercise have been proven to be advantageous for women and can decrease symptoms resulting from pregnancy.

The information presented in this systematic review could be used in a variety of settings, but primarily by healthcare providers. The interaction between pregnant women and physicians can encourage the application of proper posture exercises or workout plans to help women learn to modify and exercise correctly. This information could also promote improved techniques to help pregnant women exercise while avoiding injuries or other adverse health effects. Creating a workout plan that women could follow may help decrease the prevalence of pregnancy complications and deaths [58].

Additional studies should be performed varying the amounts of time and days between specific types of exercise to determine if variability matters. Moreover, creating a more varied exercise workout plan could improve mental health, in general, and possibly decrease postnatal depression. Alternative exercises (e.g. hiking, kayaking, etc.) should also be studied to determine possible benefits of these lesser known types of physical activities.

## Funding Info

No funding was received for this study.

## Précis

The result of this systematic review revealed that resistance training, dance, and aerobic exercises provided the most benefits during the first trimester, whereas aquatic activities and yoga helped pregnant women toward the end of their pregnancies.

## CRediT authorship contribution statement

**By Tara Rava Zolnikov:** Writing – review & editing, Writing – original draft, Supervision, Methodology, Investigation, Formal analysis, Conceptualization. **Nathalia Rodrigues-Denize:** Writing – original draft, Methodology, Formal analysis, Conceptualization. **Frances Furio:** Writing – review & editing, Methodology.

## Declaration of competing interest

The authors declare no conflicts of interest. We hereby declare that the disclosed information is correct and that no other situation of real, potential or apparent conflict of interest is known to me.

## Appendix A Supplementary data

PRISMA 2020 Flow Diagram: Pregnancy, Exercise, and Health Outcomes. Image 1

Supplementary material 2. Image 2
