# Supplementary material for: A systematic review on the physical, mental, and occupational effects of exercise on pregnant women
Source: Dialogues Health. 2024 May 12;4:100181. doi: 10.1016/j.dialog.2024.100181 (PMC11133494; doi:10.1016/j.dialog.2024.100181)
Supplement: Supplementary file 1 — PRISMA 2020 Flow Diagram: Pregnancy, Exercise, and Health Outcomes. [file mmc1.docx]

The search engines used to access scholarly articles include Google Scholar, ProQuest, EBSCOHost, PubMed, ScienceDirect, Elsevier, and the National Center for Biotechnology Information (NCBI). The Center for Disease Control and Prevention (CDC) was also utilized to uncover current statistics on pregnancy and exercise. These organizations were also used to discover prevalent pregnancy complication issues. The search strategy involved developing a grouping of terms and synonyms to determine core concepts in the review:

- Concept 1: Population e.g. pregnant women
- Concept 2: Intervention e.g. type of exercise or dancing or running or walking
- Concept 3: Population and intervention e.g. women running or women exercise

The key terms used to search the databases for suitable articles include “pregnant women and exercise or physical activity and benefits”, “pregnant women and resistance training”, “pregnant women and aquatic exercise”, “pregnant women and running”, “pregnant women and walking”, “pregnant women and yoga”, pregnant women and complications”, “pregnant women and aerobic exercise”, “exercise”, “pregnant women and physical complications”, “pregnant women and dance”, “pregnant women and cycling”, “pregnant women and dance therapy”, “pregnant women and bicycling”, and “pregnant women and mental health issues”.
