# Supplementary material for: A systematic review on the physical, mental, and occupational effects of exercise on pregnant women
Source: Dialogues Health. 2024 May 12;4:100181. doi: 10.1016/j.dialog.2024.100181 (PMC11133494; doi:10.1016/j.dialog.2024.100181)
Supplement: Supplementary file 2 — Supplementary material 2. [file mmc2.docx]

**Identification of studies via other methods**

**Identification of studies via databases and registers**

Records identified from:

Websites (n = 19)

Organisations (n = 12)

Citation searching (n =7)

etc.

Records removed *before screening*:

Duplicate records removed (n = 483)

Records marked as ineligible by automation tools (n = 264)

Records removed for other reasons (n = 90)

Records identified from*:

Databases (n = 924)

Registers (n = 47)

**Identification**

Records screened

(n = 134)

Records excluded**

(n = 7)

Reports not retrieved

(n = 21)

Reports sought for retrieval

(n = 27)

Reports sought for retrieval

(n = 127)

Reports not retrieved

(n = 32)

**Screening**

Reports assessed for eligibility

(n = 17)

Reports excluded:

Unreliable source (n = 4)

Data unavailable (n = 3)

Data to old (n = 1)

Reports assessed for eligibility

(n = 95)

Reports excluded:

Irrelevant data (n = 10)

Unable to attain (n = 5)

Publishing date too old (n = 4)

Studies included in review

(n = 76)

Reports of included studies

(n = 9)

**Included**
